# Pathophysiological Role of Caveolae in Hypertension

**DOI:** 10.3389/fmed.2019.00153

**Published:** 2019-07-10

**Authors:** Xiaoming Lian, Claudia Matthaeus, Mario Kaßmann, Oliver Daumke, Maik Gollasch

**Affiliations:** ^1^Experimental and Clinical Research Center—A Joint Cooperation Between the Charité–University Medicine Berlin and the Max Delbrück Center for Molecular Medicine in the Helmholtz Association, Berlin, Germany; ^2^Max Delbrück Center for Molecular Medicine in the Helmholtz Association, Berlin, Germany; ^3^Medical Clinic for Nephrology and Internal Intensive Care, Berlin, Germany

**Keywords:** caveolae, caveolin 1, endothelial nitric oxide synthase, Ca^2+^ channels, hypertension

## Abstract

Caveolae, flask-shaped cholesterol-, and glycosphingolipid-rich membrane microdomains, contain caveolin 1, 2, 3 and several structural proteins, in particular Cavin 1–4, EHD2, pacsin2, and dynamin 2. Caveolae participate in several physiological processes like lipid uptake, mechanosensitivity, or signaling events and are involved in pathophysiological changes in the cardiovascular system. They serve as a specific membrane platform for a diverse set of signaling molecules like endothelial nitric oxide synthase (eNOS), and further maintain vascular homeostasis. Lack of caveolins causes the complete loss of caveolae; induces vascular disorders, endothelial dysfunction, and impaired myogenic tone; and alters numerous cellular processes, which all contribute to an increased risk for hypertension. This brief review describes our current knowledge on caveolae in vasculature, with special focus on their pathophysiological role in hypertension.

## Introduction

**Figure 1:**
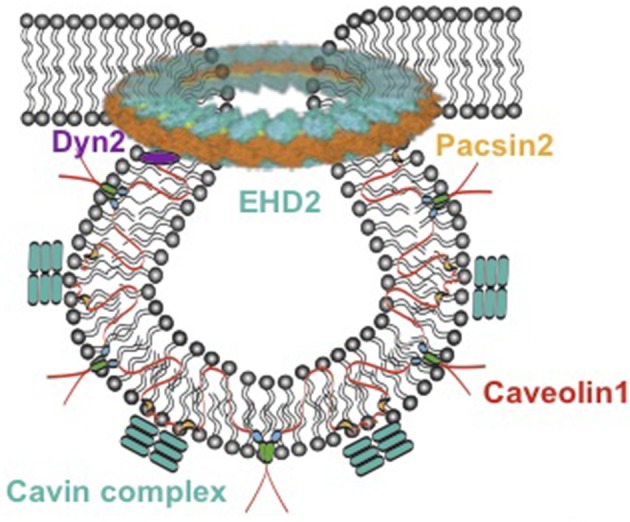
Model of caveolae. Caveolae model modified from Matthäus et al. (12). EHD2, Eps15 homology domain containing protein 2; Dyn2, Dynamin 2.

In the 1950s, 60- to 100-nm caves in the plasma membrane of the cell were first described using an electron microscope and named caveolae (1). It was later identified that most tissues and cell types contain caveolae, but the quantity varied (2). Because of the lack of experimental approaches and technologies, caveolae functions were mostly unclear until the 1990s. With the development of molecular techniques, the major membrane proteins of caveolae, caveolins, were dissected, and the secrets of function of these bulb-like caves were subsequently revealed. There are three caveolins, which are named caveolin 1 (Cav1), caveolin 2 (Cav2), and caveolin 3 (Cav3) (3). These proteins are encoded by different genes, *CAV1, CAV2*, and *CAV3*. Cav1 is expressed in most of the cell types and is essential for caveolae biogenesis; Cav3 is predominantly expressed in muscle cells (i.e., cardiac, striated skeletal, and smooth muscle cells) and is required for caveolar morphogenesis; Cav2 is generally expressed together with Cav1 in adipocytes, endothelial cells, pneumocytes, and fibroblasts, but appears to be dispensable for caveolar formation (4). Loss of either Cav1 or Cav3 results in a complete lack of caveolae (5, 6). A few years later, a series of additional proteins were identified, which play important roles in the formation of caveolae. Cavin 1–4 are essential for caveolae formation (7, 8) and function (8, 9). Together with caveolins, cavins preserve the stable coat around the bulb of caveolae (10, 11). In addition, Eps15 homology domain containing protein 2 (EHD2) is involved in mediating caveolar stabilization at the plasma membrane (Figure 1) (13); pacsin2 is a protein that is involved in the membrane bending to form caveolae as well as in mediating caveolar stabilization and scission (14, 15). Dynamin 2 is a caveolae neck-forming protein and plays a role in caveolar internalization and scission (16). Cav1 is transported from the Golgi complex to bulb from the plasma membrane and associates with cavin complex and pacsin2 to form Cav1-rich domains (11). Dynamin 2 is able to restrict the caveolar neck and EHD2 is located in this neck to stabilize the caveolae.

In accordance with major knowledge and understanding of caveolae structure, increasing interest has been focused on caveolae functions in physiology and pathophysiology. The physiological roles of caveolae vary depending on the organ systems and cell types examined (17). In the cardiovascular system, they contribute to maintaining a normal vascular tone and act as signal platforms (18–20). Signal transduction is critically important in the regulation of vascular homeostasis. Caveolae is reported to act as signaling platforms to a set of signaling molecules and receptors such as angiotensin II type 1 receptors (AT1R) (21), endothelial nitric oxide synthase (eNOS), several ion channels, and tyrosine kinase receptors (RTK) in vasculature (Figure 1) (19, 22, 23). The modification of caveolae structure affects its physiological function; e.g., alterations of lipid and proteins in caveolae induced by eicosapentaenoic acid (EPA) changed eNOS activation (24); lowering of cholesterol content in caveolae by simvastatin inhibited Akt1 serine–threonine kinase/protein kinase B (Akt/PKB) signaling pathway (25).

Loss of caveolae caused numerous vascular disorders, e.g., vascular smooth muscle hypertrophy (26), endothelial dysfunction (27), and impaired myogenic tone (28), which are all risk factors for the development of hypertension (29). A study pointed that renal hypertensive rats expressed a lower number of caveolae in aortic smooth muscle cells (SMCs) and endothelial cells, which induced an impaired effect of acetylcholine (30). Therapeutic targeting of caveolae in vascular diseases is also under study. For instance, a mutant cell-permeable scaffolding domain peptide called Cavnoxin, which can increase basal NO release in eNOS-expressing cells, has been recently identified to reduce vascular tone *ex vivo* and lower blood pressure in mice (31).

Here, we provide an overview of caveolae expression and function in the vasculature and discuss their putative role in pathophysiology of hypertension.

## Vascular Endothelial Caveolae

Electron microscopic, biochemical, and immunochemical analyses demonstrated that caveolae are highly expressed in endothelial cells (32–34). Importantly, various signaling molecules and receptors of endothelial cells enriched in caveolae, in particular eNOS (31), G-proteins (35), protein kinase A (PKA) (36), protein kinase C (PKC) (37), and various receptors (38). They have been suggested to bind and be inhibited by Cav1 through its caveolin scaffolding domain (CSD), a conserved amphipathic region for caveolae formation as well as for regulating signal transduction (35, 39, 40).

**Figure 2:**
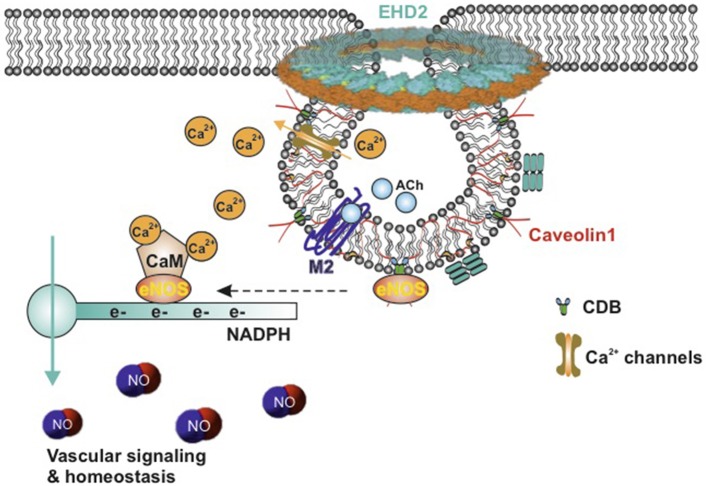
Schematic model of active eNOS in endothelial caveolae. Caveolae model modified from Matthäus et al. (12). M2-muscarinic acetylcholine receptor activation initiates an influx of Ca^2+^ that bind to calmodulin, eNOS dissociates from CBD of caveolin-1, CaM binds to eNOS, and the flow of electrons from NADPH to eNOS is restored, and then NO is produced. eNOS, endothelial nitric oxide synthase; NO, nitric oxide; CaM, calcium–calmodulin complex; CBD, caveolin scaffolding domain; ACh, acetylcholine; EHD2, Eps15 homology domain containing protein 2.

Among these caveolae-localized signaling molecules, eNOS has attracted great attention for its critical effects on vascular homeostasis and blood pressure regulation (Figure 2) (41–43). Both biochemical analysis and immunogold labeling showed that a majority of eNOS resides in caveolae of endothelial cells (44, 45). Caveolae represent a predominant location of eNOS in endothelial cells (45, 46). The studies emphasize a critical role of endothelial caveolae in regulating activation of eNOS (Figure 2). In inactive endothelial cells, eNOS is shown to associate with Cav1, which inhibits calmodulin complex (CaM) binding to eNOS (47). This combination interrupts the electrons from NADPH to eNOS. M2-muscarinic acetylcholine receptor activation or other stimulation (e.g., increasing vascular flow and pressure) initiates an influx of Ca^2+^ that binds to calmodulin. In succession, eNOS dissociates from Cav1 and then combines to CaM. The flow of electrons from NADPH is therefore restored and consequently NO is produced (Figure 2) (48, 49). This NO generation results in the association of Cav1 and eNOS as previously shown, thus terminating the signal transduction (32). Furthermore, due to the increased cytosolic Ca^2+^ concentration, eNOS translocated from the cell membrane to the Golgi complex and is fully activated. After all, eNOS localizes back to the plasma membrane (35, 50, 51). Moreover, it has been demonstrated *in vivo* and *in vitro* that caveolin 1 is able to bind eNOS and therefore to inhibit the synthesis of NO (47, 52, 53). Increased expression of Cav1 is known to appear in patients with insulin resistance and type 2 diabetes (54), associated with impaired acetylcholine-induced NO production and vasodilation (55). Cav1 knockout mice show chronic and dramatic elevation in systemic NO levels and enhanced acetylcholine-induced arterial relaxation (56, 57). Conversely, the Bendhack group (58) reported that caveolae disassembled by methyl-β-cyclodextrin (mβcd) treatment cause an impaired acetylcholine-induced relaxation in the rat isolated aorta. This is in agreement with another study showing that caveolar disruption results in a decreased release of endothelial-derived NO in femoral arteries (59). This difference is supposed to be due to chronic Cav1 deficiency vs. acute caveolar disruption, as upon chronic lack of Cav1, an adjustment of an attenuated myogenic tone can be observed over a longer time period, i.e., active constriction induced by pressure, as compensation at the level of the vascular wall (60, 61).

The elevation of systemic NO levels and impaired myogenic tone in Cav1 knockout mice would be expected to show a lower systemic blood pressure (6, 28). However, there are several studies reporting no difference in systolic and diastolic blood pressure between Cav1 knockout and wild-type mice (62–64). Of note, Wunderlich et al. (65) reported reduced systemic blood pressure whereas Pojoga et al. (66) reported an elevated systolic blood pressure in Cav1 knockout mice. Another study showed that Cav1 knockout mice have a slightly increased heart rate (60), suggesting possible compensation via increased baroreceptor reflex activation leading to increased sympathetic activity and neurogenic tone. Moreover, what should also be mentioned is that the deletion of Cav1 impaired the Mg^2+^ absorption and increases K^+^ excretion in renal distal convoluted tubule (67). The involvements of baroreceptor reflex and electrolytes disturbance in blood pressure regulation of Cav1 knockout mice make this scenario even more complicated.

Last but not least, endothelial transient receptor potential vanilloid receptor 4 (TRPV4) channels, where the NO-dependent vasodilation in arteries is triggered by Ca^2+^ entry (68), colocalize with Cav1 in the caveolae-enriched membrane fractions (69). These channels are potent Ca^2+^ influx channels (Figure 2). Cav1 knockout results in total absence of TRPV4-induced relaxation, suggesting that caveolae are essential for TRPV4 function and Ca^2+^ signaling in endothelial cells (68).

## Vascular Smooth Muscle Caveolae

Caveolae are also abundant in SMCs, which are known to express Cav1–3 (70). Similar to the endothelial cells, caveolae are also important for SMC function, providing a platform for signal transductions through G-protein-coupled receptors and ion channels, therefore helping to maintain vascular homeostasis (18).

**Figure 3:**
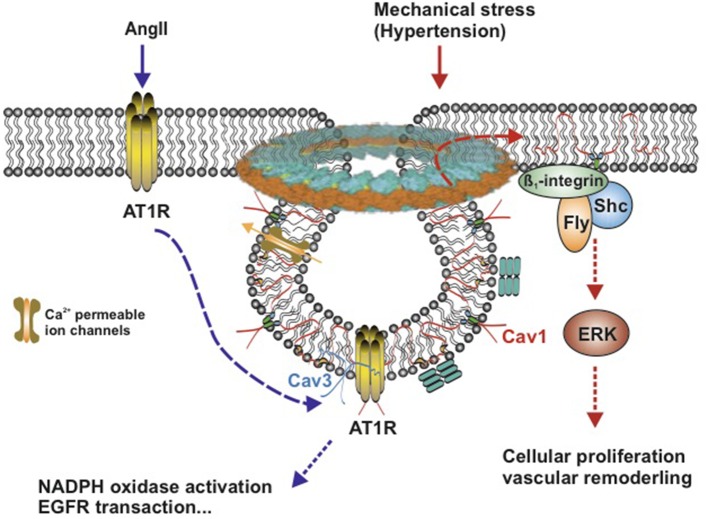
Schematic model of AngII-induced and mechanical stress-induced signaling pathway in vascular smooth muscle caveolae. Caveolae model modified from Matthäus et al. (12). **Left:** Ang II induces rapid translocation of AT1R to caveolae, AT1R and Caveolin 1 associate with each other. Caveolin 3 accompanies with AT1R. **Right:** Mechanical stress induces translocation of Caveolin 1 to non-caveolar sites and is associated with β_1_-integrins/Fyn/Shc to activate ERK signaling pathway. Cav1, Caveolin 1; Cav3, Caveolin 3; AngII, Angiotensin II; AT1R, AngII type1 receptor; ERK, extracellular signal-regulated kinase; Ca^2+^-permeable ion channels, such as TRPC, transient receptor potential channels, and EGFR, epidermal growth factor receptor.

Angiotensin II (Ang II) working through AT1R is a well-known signaling pathway in vascular SMCs, which plays a great role in renal hypertension. Dysfunction of this pathway shows a predominant role in the pathophysiology of renal hypertension and several renal diseases (71, 72). In vascular SMCs, Ang II induces rapid translocation of a subset of AT1Rs to caveolae, where AT1Rs bind to Cav1 (73, 74) (Figure 3), which, in succession, activate the downstream signaling events, such as NADPH oxidase activation (cell migration and growth) (75), Ca^2+^ mobilization (arterial contractile responses) (6), epidermal growth factor receptor (EGFR) transactivation (tyrosine phosphorylation) (73, 76), and vascular SMC hypertrophy (77). Cav1 showed a beneficial effect in hypertensive mice. Cav1 protected against the development of systemic high blood pressure and enhanced resistance artery constriction through its binding to AT1R, which delays AT1R reactivation after Ang II stimulation (78). Ang II-induced hypertensive vascular remodeling is attenuated in Cav1 knockout mice (79).

Besides endogenous hormones, hypertension-induced mechanical stress contributes to the genesis of vascular hypertrophy and vascular remodeling, which can induce translocation of Cav1 in vascular SMCs (80). In response to chronic shear stress, Cav1 is translocated to non-caveolar sites and then combined to β_1_-integrins/Fyn/Shc, which mediates stretch-induced extracellular signal-regulated kinase (ERK) activation (80–82) (Figure 3). When exposed to acute mechanical stresses, caveolae disassemble completely and rapidly lead to the translocation of caveolins in the plasma membrane (<30 s), which flattens out caveolae in the plasma membrane to provide additional membrane and extra buffer tension (83). This reversible and rapid disassembly of caveolae provides a basic vascular response to an acute shear stress (Figure 3).

In vascular smooth muscle, most of the physiological processes are known to require Ca^2+^ (84). Ca^2+^ flux and intracellular Ca^2+^ level take part in numerous physiological processes of smooth muscle (85). Vascular caveolae are known to provide functional organization of ion channels, in particular calcium channels. Although multiple Ca^2+^ handling molecules [the plasma membrane Ca^2+^ pump (PMCA) (86), Na^+^-Ca^2+^ exchanger (NCX1) (87), T-type Ca_v_3.2 channels (88), and transient receptor potential canonical channels (TRPCs) (89) are shown to localize or associate with Cav1 or Cav3 within caveolae (Figure 3); the exact function of most Ca^2+^ handling molecules in vascular SMCs regarding intracellular Ca^2+^ signaling remains elusive (90).

**Figure 4:**
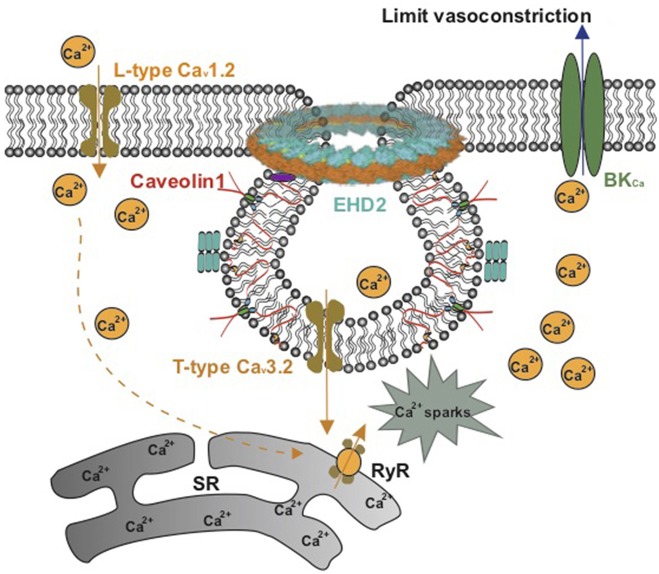
Schematic model on the role of caveolae in Ca^2+^ signaling in vascular smooth muscle. L-type Ca_v_1.2 channels in noncaveolar membrane sites and T-type Ca_v_3.2 channels in caveolae produce Ca^2+^ influx into vascular SMCs to release Ca^2+^ sparks *via* ryanodine type 2 receptors (RyR) in the sarcoplasmic reticulum (SR). The Ca^2+^ sparks produce a negative-feedback effect on vasoconstriction by activating maxi Ca^2+^-activated K^+^ (BK_Ca_) channels.

Myogenic tone, which serves to regulate blood flow and protect downstream vessels from pressure-induced damage, is largely dependent on an influx of extracellular Ca^2+^ via voltage-operated calcium channels (91). In large cerebral vessels *in vitro*, the myogenic tone is mainly regulated by L-type Ca_v_1.2 channels (92). However, as the vessel size decreases (<40 μm), L-type Ca_v_1.2 channels have been reported to disappear (93). Recent studies showed that T-type Ca_v_3.2 channels functionally located in caveolae activate BK_Ca_ channels to limit vasoconstriction (88, 94–96). This spatial functional organization between T-type Ca_v_3.2 channels, ryanodine receptors, and BK_Ca_ channels contrasts the role of L-type Ca_v_1.2 channels in non-caveolar membrane sites to produce primary Ca^2+^ influx into vascular SMCs and release of Ca^2+^ sparks *via* indirect ryanodine type 2 receptor (RyR) activation through sarcoplasmic reticulum Ca^2+^ content (88, 97–99) (Figure 4). In both cerebral and mesenteric arteries, T-type calcium currents show increased amplitudes as vessel size decreases (93, 100). Genetic deletion of Cav1 or mβcd treatment of vascular SMCs impairs caveolae formation and impacts either the activity or localization of T-type Ca_v_3.2 channels (88, 95, 101). Together, the data support the idea that T-type Ca_v_3.2 channels within caveolae play an important role in the regulation of myogenic tone in small peripheral resistance vessels, which may represent an attractive explanation for attenuated myogenic arterial tone observed in Cav1 knockout models.

Another remarkable ion channel group located in vascular SMC caveolae are TRPC channels, which are suggested to work as store-operated Ca^2+^ entry (SOCE) channels and are essential for the restoration of internal Ca^2+^ (102). As SOCE channels, TRPC channel proteins need to associate with other regulatory signaling molecules within caveolae, where they provide a platform for the assembly of TRPC signalplex, including Cav1, G protein, and G-protein-coupled receptor (103). Cav1 deficiency reduced agonist-stimulated Ca^2+^ secretion and disrupted TRPC signalplex assembly (104). TRPC/TRPC signalplex shows an important role not only in the pathogenesis of pulmonary hypertension (105, 106) but also in essential hypertension and renal hypertension (107–109), which indicated that TRPC or TRPC signalplex may act as important new targets for treatment of hypertension.

## Perspective and Conclusions

Caveolae, cholesterol- and glycosphingolipid-rich membrane microdomains, serve as a platform for signal transduction in endothelial cells and vascular SMCs. Within the caveolae membrane domain, in particular Cav1, is a critical molecule, allowing for the rapid activation by posttranslational protein modification. Deletion of caveolin genes is not lethal but caveolin knockout mice show several vascular disorders and dysfunction (110–113) (Table 1).

**Table 1:** Molecules located in caveolae and their effects in Cav 1 deficient mice in vasculature.

**Molecules located in caveolae**	**Deletion of caveolin genes associated with**	**References**
**VASCULAR ENDOTHELIAL CELLS**		
eNOS	Elevated NO levels	(55, 56)
	Enhanced vasodilation	(55, 56)
	Impaired vasodilation	(57, 59, 60)
	Decreased release of NO	(58–60)
	Impaired myogenic tone	(6, 27, 60)
	Unchanged blood pressure	(61–63)
	Reduced blood pressure	(64)
	Elevated blood pressure	(65)
TRPV4	Impaired TRPV4-induced relaxation	(67, 68)
**VASCULAR SMOOTH MUSCLE CELLS**		
AngII	Enhanced vascular remodeling	(78)
Ca^2+^ channels	Elusive effects	(89)
T-type Ca_v_3.2 channels	Attenuated myogenic tone	(87, 94, 100)
TRPC	Reduced agonist-stimulated Ca^2+^ secretion	(103)
	Hypertension	(104–108)

*eNOS, endothelial nitric oxide synthase; NO, nitric oxide; TRPV4, transient receptor potential vanilloid receptors 4; AngII, Angiotensin II; TRPC, transient receptor potential canonical*.

In this review, we discussed pathophysiological roles of caveolae in endothelial and vascular SMCs in hypertension, although caveolae most likely also have an impact on vascular function in different tissues like adipose tissue. In the vasculature, except cerebral arteries, blood vessels are directly surrounded by perivascular adipose tissue (PVAT), which directly expresses and secretes Cav1 (18, 113, 114). Numerous studies point out a putative role of caveolae in adipocytes in regulating lipid trafficking, storage, and modulating insulin signal transduction and metabolism (115–118). Moreover, EHD2, which is located in the neck of caveolae, seems to act as a negative regulator of caveolae-dependent lipid uptake (12). These data suggest that adipocyte-secreted Cav1 and EHD2 could contribute to vascular metabolic diseases; however, direct evidence is missing and remains to be determined.

An increasing number of caveolae-associated diseases are explored in order to achieve a more precise assessment of caveolae function in various pathophysiological conditions in vasculature. With the convinced and diversified roles of caveolae in the vasculature, studies on future therapeutic targeting of caveolae in hypertension are necessary.

## Author Contributions

All authors listed have made a substantial, direct and intellectual contribution to the work, and approved it for publication.

### Conflict of Interest Statement

The authors declare that the research was conducted in the absence of any commercial or financial relationships that could be construed as a potential conflict of interest.
